# Functional and prognostic relevance of the homeobox protein MSX2 in malignant melanoma

**DOI:** 10.1038/bjc.2011.249

**Published:** 2011-07-05

**Authors:** G Gremel, D Ryan, M Rafferty, F Lanigan, S Hegarty, M Lavelle, I Murphy, L Unwin, C Joyce, W Faller, E W McDermott, K Sheahan, F Ponten, W M Gallagher

**Affiliations:** 1 UCD School of Biomolecular and Biomedical Science, UCD Conway Institute, University College Dublin, Belfield, Dublin 4, Ireland; 2 OncoMark Ltd, NovaUCD, Belfield, Dublin 4, Ireland; 3 UCD School of Medicine and Medical Science, UCD Conway Institute, University College Dublin, Belfield, Dublin 4, Ireland; 4 Whitla Medical Building, Queen's University Belfast, Lisburn Road, BT9 7BL Belfast, UK; 5 Department of Pathology, St Vincent's University Hospital, Elm Park, Dublin 4, Ireland; 6 Department of Genetics and Pathology, Rudbeck Laboratory, Uppsala University Hospital, SE-751 85 Uppsala, Sweden

**Keywords:** melanoma, MSX2, homeobox, apoptosis, spheroid invasion, biomarker

## Abstract

**Background:**

The homeobox containing transcription factor MSX2 is a key regulator of embryonic development and has been implicated to have a role in breast and pancreatic cancer.

**Methods:**

Using a selection of two- and three-dimensional *in vitro* assays and tissue microarrays (TMAs), the clinical and functional relevance of MSX2 in malignant melanoma was explored. A doxycyline-inducible over-expression system was applied to study the relevance of MSX2 *in vitro*. For TMA construction, tumour material from 218 melanoma patients was used.

**Results:**

Ectopic expression of MSX2 resulted in the induction of apoptosis and reduced the invasive capacity of melanoma cells in three-dimensional culture. MSX2 over-expression was shown to affect several signalling pathways associated with cell invasion and survival. Downregulation of N-Cadherin, induction of p21 and inhibition of both BCL2 and Survivin were observed. Cytoplasmic MSX2 expression was found to correlate significantly with increased recurrence-free survival (*P*=0.008). Nuclear expression of MSX2 did not result in significant survival correlations, suggesting that the beneficial effect of MSX2 may be independent of its DNA binding activity.

**Conclusions:**

MSX2 may be an important regulator of melanoma cell invasion and survival. Cytoplasmic expression of the protein was identified as biomarker for good prognosis in malignant melanoma patients.

Homeobox proteins are transcriptional regulators that occupy a crucial role during development and are frequently found to be aberrantly expressed in cancer (Abate-Shen, 2002). *MSX* genes in vertebrates are related to the *Drosophila* muscle segment homeobox (Msh) protein. In man, two members of this family, MSX1 and MSX2, have been described (Hewitt *et al*, 1991; Jabs *et al*, 1993). MSX2 has a crucial role during the development of multiple organ systems (Satokata *et al*, 2000). Next to a complex role in the regulation of cell differentiation and proliferation, MSX2 expression is frequently associated with the induction of apoptosis. Recently, over-expression of MSX1 and MSX2 has been shown to be, in part, responsible for an excess of apoptosis in the developing limbs of sonic hedgehog-1 (SHH1) knockout mice (Lallemand *et al*, 2009). It is proposed that, in the limb, *MSX* genes are downstream regulators of the SHH/GLI3 pathway through transduction of bone morphogenetic protein (BMP) signalling. Similarly, MSX2 over-abundance has been shown to induce apoptosis in numerous other cell types including capillary endothelial cells in the pupillary membrane (Kiyono and Shibuya, 2003) and premigratory neural crest cells (Takahashi *et al*, 1998).

With regard to studies in tumour model systems, we recently showed induction of apoptosis in both human breast immortalised and transformed cell lines in response to MSX2 over-expression (Lanigan *et al*, 2010). A more refined impact of ectopic MSX2 expression has been described in murine P19 embryonal carcinoma cells, where increased levels of apoptosis were only apparent upon cell aggregation but could not be detected when cells were grown in a monolayer (Marazzi *et al*, 1997). Together with reports on the necessity of tissue interactions for sustained expression of MSX1 and MSX2 (Davidson, 1995), this points towards a highly specific role of MSX2 within the complex biological apparatus of cell signalling pathways. In the developing embryo, MSX2 expression is not only confined to areas of programmed cell death but also frequently detected at sites of epithelial–mesenchymal interactions (Davidson, 1995). Moreover, over-expression of MSX2 in human pancreatic cancer cells (Satoh *et al*, 2008), as well as in mouse mammary epithelial cells (di Bari *et al*, 2009), has been shown to induce epithelial-to-mesenchymal transition (EMT). This highlights a diverse, cell type specific, functional role of MSX2.

Examination of the clinical relevance of MSX2 in tumourigenesis and patient outcome has only been performed in a handful of studies with mostly limited sample sizes. In a study of 32 pancreatic cancer patients, high levels of MSX2 significantly correlated with higher tumour grades and vascular invasion (Satoh *et al*, 2008). In a follow-up study on 45 patients with branch duct intraductal papillary-mucinous neoplasia of the pancreas, MSX2 expression was shown to gradually increase from benign to malignant lesions (Satoh *et al*, 2010). A comprehensive, large-scale study on breast cancer, however, recently associated the expression of MSX2 with favourable disease characteristics (Lanigan *et al*, 2010). Analysis of a publicly available transcriptomic data set derived from 295 primary invasive breast tumours (van de Vijver *et al*, 2002) pointed towards a role of MSX2 as marker for good prognosis. This was confirmed at the protein level, where tissue microarray (TMA)-based analysis of 281 invasive breast carcinomas showed a significant correlation of cytoplasmic MSX2 expression with low-grade tumours and prolonged patient survival (Lanigan *et al*, 2010).

Here, the functional and clinical relevance of MSX2 for malignant melanoma is explored. Ectopic expression of MSX2 in melanoma cell lines led to the induction of apoptosis and inhibited cell invasion in a three-dimensional spheroid assay. Using TMA technology, we found that cytoplasmic expression of MSX2 was significantly associated with improved recurrence-free and overall survival of melanoma patients.

## Materials and methods

### Cell culture

WM793 and 1205Lu cells were obtained from the European Searchable Tumour Line Database (ESTDAB, Tübingen, Germany). They were maintained in RPMI Medium 1640-GlutaMAX-I (Invitrogen, Carlsbad, CA, USA) supplemented with 10% (v/v) fetal calf serum, 50 IU ml^−1^ penicillin and 50 *μ*g ml^−1^ streptomycin sulphate. SKBR3 and MDA-MB-231 cells were purchased from the European Collection of Cell Cultures (Wiltshire, UK) and grown in DMEM (Sigma-Aldrich, St Louis, MO, USA) supplemented with 10% (v/v) fetal calf serum, 2 mM L-glutamine, 50 IU ml^−1^ penicillin and 50 *μ*g ml^−1^ streptomycin sulphate. All cell lines tested negative for mycoplasma contamination.

### MSX2 over-expression

Subconfluent HEK293T cells were transfected with LLCIEP vector (Trono Laboratories, Lausanne, Switzerland) without insert or containing full-length MSX2 cDNA (including a V5 tag) or with pLV-tTR-KRAB (KRAB) vector (Trono Laboratories), together with packaging (psPAX2) and envelope vectors (pMD2G) (both from Trono Laboratories) via calcium phosphate precipitation. After 7 h, the media was refreshed and 48 h later recombinant lentivirus harvested by passing the culture media through a 0.45-μm low protein binding filter. Melanoma cells were infected by adding a mixture containing one part MSX2 over-expression construct or empty vector encoding virus, one part KRAB encoding virus and one part fresh media. After 48 h, this mixture was replaced with fresh media. To induce the expression of MSX2, doxycycline (DOX) at a final concentration of 0.05 *μ*g ml^−1^ (or as specified) was added to the culture medium. For western blot and immunofluorescence analysis, cells were induced 6 days before protein isolation or staining. For functional assays, cells were pre-induced for 3 days and DOX treatment maintained throughout the duration of the assay (unless indicated differently).

### Western blot and immunofluorescence analysis

Protein preparations were separated by SDS–PAGE and blotted onto PVDF membranes. After blocking with 5% milk in Tris-buffered saline containing 0.1% Tween-20 (TBS-T), primary antibody incubation took place overnight at 4 °C. The following antibodies were used: MSX2 (Clone 2E12, Abcam, Cambridge, UK); N-Cadherin (Clone 32, BD Biosciences, San Jose, CA, USA); BCL2 (Clone C-2, Santa Cruz Biotechnology, Santa Cruz, CA, USA); Survivin (Clone D-8, Santa Cruz Biotechnology); p21 (Clone SX118, BD Biosciences); AKT (Clone 9272, Cell Signaling Technology, Danvers, MA, USA), p-AKT (Ser473, Clone T308, Cell Signaling Technology), ERK1 (Clone C-16, Santa Cruz Biotechnology), p-ERK1 (Clone E-4, Santa Cruz Biotechnology) and *β*-Actin (Clone 11B7, Santa Cruz Biotechnology). For signal detection, species-specific horseradish peroxidase (HRP)-conjugated secondary antibodies and Enhanced Chemiluminescence Plus Substrate (Amersham Biosciences, Buckinghamshire, UK) were applied, followed by autoradiography using X-ray film. Immunofluorescence studies were performed as described previously (Lanigan *et al*, 2010).

### Two-dimensional *in vitro* characterisation

Two-dimensional *in vitro* assays have previously been described in detail (Lanigan *et al*, 2010). Briefly, for 3-(4,5-dimethylthiazol-2-yl)-2,5-diphenyltetrazolium bromide (MTT) assays, 5000 cells per well were seeded into 96-well plates and assayed over six consecutive days. Statistical significance was generated from signal changes over consecutive days, respectively. For colony formation assays, 500 cells per well were seeded into 6-well plates and colonies counted after 3 weeks. The Caspase-Glo 3/7 assay was purchased from Promega (Madison, WI, USA) and used according to the manufacturer's instructions. In all, 5000 cells per well were seeded into a black walled 96-well plate and assayed 3 days later. To normalise the luminescent signal to the number of cells present, a second plate was set up simultaneously and cells per well counted using a Beckman Coulter Z1 Coulter Counter (Beckman Coulter, Brea, CA, USA).

### Cell-cycle analysis

Cells were DOX-induced for 6 days, detached using trypsin, washed with phosphate-buffered saline (PBS) and fixed with 70% (v/v) ethanol in PBS. Fixed cells were centrifuged, washed with PBS and resuspended in PBS containing 20 *μ*g ml^−1^ propidium iodide (PI) and 60 *μ*g ml^−1^ RNAse A (both from Sigma-Aldrich). After 30 min incubation at room temperature in the dark, cells were analysed on a C6 Flow Cytometer System (Accuri Cytometers, Ann Arbour, MI, USA).

### Spheroid invasion assay

Five thousand cells per well were seeded into 96-well plates coated with 1.5% agarose in PBS. Cells were induced at the time of seeding. After 3 days, spheroids were harvested using a P1000 pipette and transferred into 3.5% bovine collagen type I (Invitrogen) made up in 1 × MEM supplemented with 2% (v/v) fetal calf serum, 50 IU ml^−1^ penicillin, 50 *μ*g ml^−1^ streptomycin sulphate and 0.05 *μ*g ml^−1^ DOX, where applicable. Normal growth media containing 2% (v/v) fetal calf serum was layered on top. After 3 days, the top media was replaced with 1 ml of PBS containing 4 *μ*g ml^−1^ Calcein-AM and 12 *μ*g ml^−1^ PI. After 1 h incubation at 37 °C in the dark, spheroids were imaged under × 4 magnification using an Andor Revolution spinning disc laser microscopy system (Andor, Belfast, Northern Ireland). Images were taken from the plane with highest visible cell dispersion using excitation wavelengths of 488 nm (Calcein-AM) and 560 nm (PI). Grey scale images for both channels were converted into .jpg file format using ImageJ software (http://rsbweb.nih.gov/ij/) and analysed on Aperio ImageScope software version 9 (Aperio Technologies, Vista, CA, USA) (Supplementary Figure 1). Briefly, using images derived from the 488 nm channel, the central area of the spheroids was marked and measured using the negative pen tool. Subsequently, an area of interest was drawn around the entire structure and, through application of a positive pixel count algorithm, the area of cell invasion determined. The latter was normalised to the radius of the spheroid core, as shown in Supplementary Figure 1. The percentage of dead cells was analysed by applying a positive pixel count algorithm within the area of the spheroid core using images taken in the 560 nm channel.

### Cell pellet arrays

Subconfluent cells were detached using trypsin and fixed for 4 h in 10% (v/v) phosphate-buffered formalin. After centrifugation, cells were resuspended in 1% agarose and processed in a Leica TP1020 tissue processor (Leica Microsystems, Wetzlar, Germany). The cell pellets were embedded in paraffin and further treated as described below (see TMA construction).

### Patients and clinical material

Formalin-fixed, paraffin-embedded (FFPE) tumour material derived from a cohort of 218 patients diagnosed with primary cutaneous melanoma at the St Vincent's University Hospital, Dublin, between 1994 and 2007 was used for TMA construction. Histopathological information could be retrieved for 205 patients (Supplementary Table 1). Follow-up data regarding overall and recurrence-free survival were available for 179 patients. The study has been reviewed and ethical approval granted by the Medical Ethics Board of St Vincent's University Hospital.

### TMA construction

Haematoxylin and eosin (H&E) stained full-face sections of all samples were reevaluated by a pathologist and tumour areas were marked. The TMA was constructed using a Beecher Manual Tissue Arrayer I (Beecher Instruments Inc., Sun Prairie, WI, USA). Four 1.0 mm tissue cores were extracted from each donor block and placed into recipient paraffin blocks. Immunohistochemistry (IHC) was carried out on 5 *μ*m sections.

### Immunohistochemistry

The TMA and cell pellet array (CPA) sections were rehydrated in descending gradient alcohols before heat-mediated antigen retrieval in Tris-HCl Buffer For Heat-Induced Epitope Retrieval (LabVision, Thermo Fisher Scientific, Waltham, MA, USA) at 95 °C for 15 min. Immunohistochemistry for MSX2 (Clone 2E12, Abcam) was performed in a LabVision Autostainer 360 (LabVision, Thermo Fisher Scientific) using an Ultra Vision LB Detection System Large Volume AP Polymer kit (LabVision, Thermo Fisher Scientific). A mouse IgG isotype control (Vector Laboratories, Burlingame, CA, USA) was used to assure antibody specificity (data not shown). To visualise antibody binding, the Vector Red Alkaline Phosphatase Substrate Kit I (Vector Laboratories) was used. After counterstaining in haematoxylin, slides were mounted manually in 50% glycerol and scanned at × 20 magnification using a ScanScopeXT slide scanner (Aperio Technologies).

### Statistical analysis

The *χ*^2^-test was used to test for associations between MSX2 expression and histopathological parameters. Kaplan–Meier blots were generated to evaluate links with patient survival and the log rank test used to compare curves separated according to MSX2 expression. Cox proportional hazards regression was used to estimate hazard ratios in both univariate and multivariate models. All calculations were performed in SPSS version 15 (SPSS Inc., Chicago, IL, USA). The significance of functional assays was determined through application of unpaired, two-tailed Student's *t*-tests. All results represent at least three independent replicates.

## Results

### MSX2 over-expression induces apoptosis in melanoma cell lines

**Figure 1:**
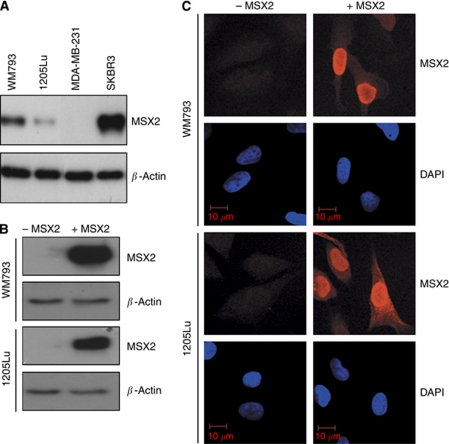
Evaluation of endogenous MSX2 protein expression in WM793 and 1205Lu cells and confirmation of DOX-induced MSX2 over-expression. (**A**) Western blot analysis of MSX2 protein expression in WM793 and 1205Lu cells. MSX2 migrated at ∼37 kDa. *β*-Actin levels were used to assess equality of protein loading. The breast cancer cell lines SKBR3 and MDA-MB-231 served as positive and negative controls for the expression of MSX2, respectively. (**B**) Western blot analysis and (**C**) immunofluorescent staining for MSX2 in cell lines with conditional MSX2 expression. −MSX2, non-induced cells; +MSX2, DOX-induced cells.

Initially, the expression of MSX2 in the melanoma cell lines WM793 and 1205Lu was tested via western blot analysis (Figure 1A). 1205Lu is an isogenic derivative of WM793, isolated from a spontaneous lung metastasis after subcutaneous injection of WM793 cells into a nude, immunodeficient mouse (Juhasz *et al*, 1993). 1205Lu cells display increased invasive capacity *in vitro* and tumourigenic capacity *in vivo* (Gallagher *et al*, 2005; Smalley *et al*, 2006). The breast cancer cell lines, SKBR3 and MDA-MB-231, served as positive and negative controls for MSX2 expression, respectively (Lanigan *et al*, 2010). Downregulation of MSX2 expression was observed in 1205Lu cells, as compared with the parental WM793 cell line. However, both melanoma cell lines expressed low MSX2 levels compared with SKBR3. Regarding the functional diversity of MSX2 in breast and pancreatic cancer (Satoh *et al*, 2008; Lanigan *et al*, 2010), DOX-inducible MSX2 over-expression systems (Wiznerowicz and Trono, 2003) were established in both WM793 and 1205Lu cells. Empty vector/tTR-KRAB transduced control cells, with/without DOX treatment, were included in all assay runs and behaved similar to the non-induced MSX over-expression cell line (Supplementary Figure 2). Successful MSX2 over-expression was confirmed via western blot analysis (Figure 1B) and immunofluorescent staining (Figure 1C). Ectopically expressed MSX2 was located primarily to the nucleus but also to the cytoplasm of over-expressing cells.

**Figure 2:**
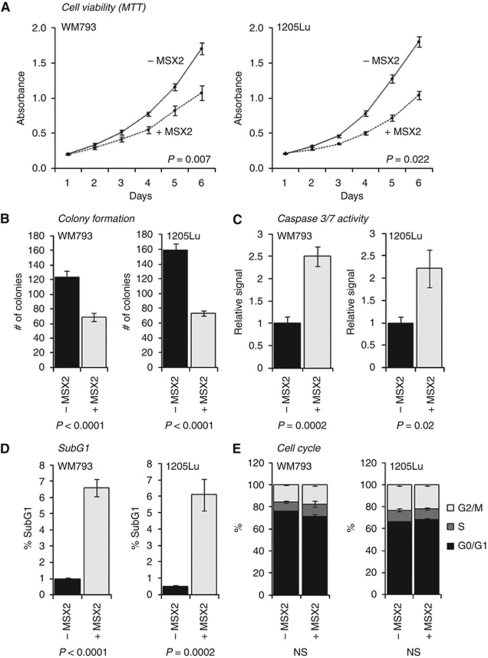
Functional characterisation of DOX-induced (+MSX2) *vs* non-induced control cells (−MSX2) in two-dimensional cultures of WM793 and 1205Lu cells. (**A**) Evaluation of cell viability over time using MTT assays. Significance was generated from the average signal change between consecutive days in three independent experiments. (**B**) Determination of clonogenic cell survival. (**C**) Assessment of Caspase 3/7 activation using a bioluminescence-based Caspase-Glo 3/7 assay system. Luminescence readings were normalised to the total cell number and displayed relative to non-induced control cells. (**D**) SubG1 and (**E**) G0/G1, S and G2/M phases of the cell cycle as determined via PI staining and flow cytometric analysis. (For representative histograms, see Supplementary Figure 3.) All readings represent the average of three independent replicates. Error bars represent the standard error of the mean within all replicate experiments. Significance levels were determined using an unpaired, two-tailed Student's *t*-test. NS, not significant.

To assess cell viability in response to MSX2 over-expression, a series of MTT assays was performed over six consecutive days (Figure 2A). MSX2-induced cells showed a significant reduction in cell viability over time in both WM793 and 1205Lu cells (*P*=0.007 and *P*=0.022, respectively). Similarly, using clonogenic assays, a significant decrease in the ability to form colonies was observed in cells over-expressing MSX2 (*P*<0.0001 in both WM793 and 1205Lu cells; Figure 2B). To investigate whether both phenotypes could be attributed to an onset of cell death mediated by apoptosis, a bioluminescence-based assay for the detection of Caspase 3/7 activation (Caspase-Glo 3/7) was performed (Figure 2C). In both WM793 (*P*=0.0002) and 1205Lu (*P*=0.02) cells, a significant increase of Caspase 3/7 activity in response to MSX2 over-expression was detected. To establish a possible impact of MSX2 on the progression of the cell cycle, cells were stained with PI and analysed using flow cytometry. In concordance with the previously described, pro-apoptotic role of MSX2, a significant increased in the SubG1 population of the cell cycle was detected in WM793 (*P*<0.0001) and 1205Lu cells (*P*=0.0002) in response to MSX2 over-expression (Figure 2D; Supplementary Figures 3A and C). Other cell-cycle phases failed to show significant changes in response to DOX-induced MSX2 expression (Figure 2E; Supplementary Figures 3B and D). The effect of MSX2 over-expression on cell viability was shown to be dose dependent (Supplementary Figure 4).

### MSX2 over-expression inhibits spheroid invasion

**Figure 3:**
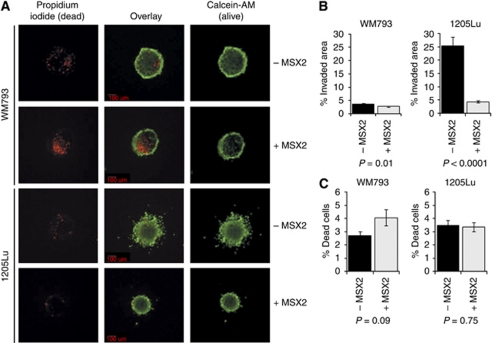
Evaluation of the impact of DOX-induced MSX2 expression on the invasive capacity of 1205Lu cells in a three-dimensional spheroid invasion assay. (**A**) Representative fluorescent images of collagen implanted spheroids. Images were taken in × 4 magnification from the plane with highest visible cell dispersion of each spheroid using a spinning disc confocal microscope. Viable cells were stained with the green fluorescent dye, Calcein-AM, while dead cells appeared red upon PI incorporation. (**B**) Numerical analysis of invaded area as detailed in Supplementary Figure 1. (**C**) Assessment of the percentage dead cells relative to the spheroid area.

To study a possible involvement of MSX2 in the regulation of melanoma cell invasiveness, a three-dimensional spheroid invasion assay was adopted. Cells were grown without anchorage until they formed globular, multi-cellular clusters that subsequently were implanted into bovine collagen type I and left to invade for 3 days. After staining with Calcein-AM (live cell stain) and PI (dead cells), the spheroids were imaged and results analysed using Aperio ImageScope software (Supplementary Figure 1). MSX2 over-expression resulted in a marked decrease in invasion (Figures 3A and B). This was particularly apparent in the invasive cell line 1205Lu, where ectopic expression of MSX2 led to an almost six-fold decrease in cell invasion (*P*<0.0001). Interestingly, for WM793 cells, only a non-significant and, for 1205Lu cells, no increase in the percentage of dead cells could be observed in this assay (Figure 3C).

### MSX2 over-expression impacts signalling pathways

**Figure 4:**
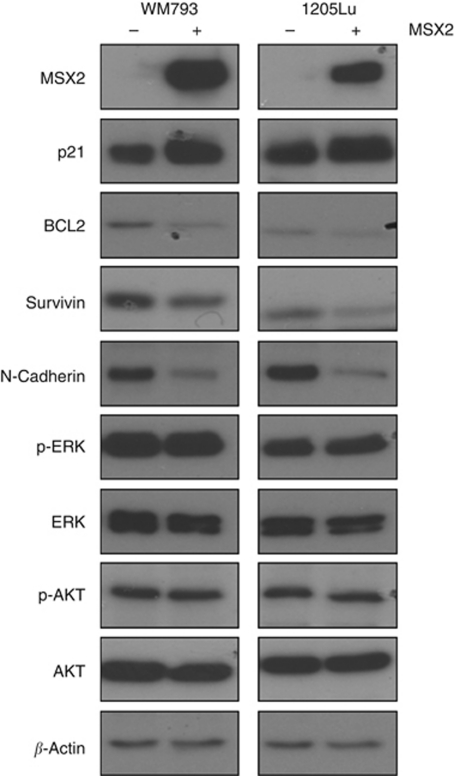
Western blot analysis of proteins involved in cell survival and invasion in response to MSX2 over-expression (+MSX2) compared with non-induced cells (−MSX2) in WM793 and 1205Lu cells. Images are representative of three independent protein extracts.

In order to investigate the molecular mechanisms by which MSX2 might regulate apoptosis and cell invasion, the expression of several proteins involved in respective signalling pathways was examined (Figure 4). For both WM793 and 1205Lu cells, a distinct downregulation of N-Cadherin was observed in response to MSX2 induction. At the same time, E-Cadherin expression remained undetectable (data not shown). The cyclin-dependent kinase inhibitor p21 (CIP1/WAF1), a protein associated with BMP-induced apoptosis (Gomes and Kessler, 2001; Israsena and Kessler, 2002), showed noticeable upregulation, while the inhibitors of apoptosis, BCL2 and Survivin, were markedly reduced in their expression. ERK and AKT signalling remained unchanged.

### Immunohistochemical detection of MSX2 protein expression

**Figure 5:**
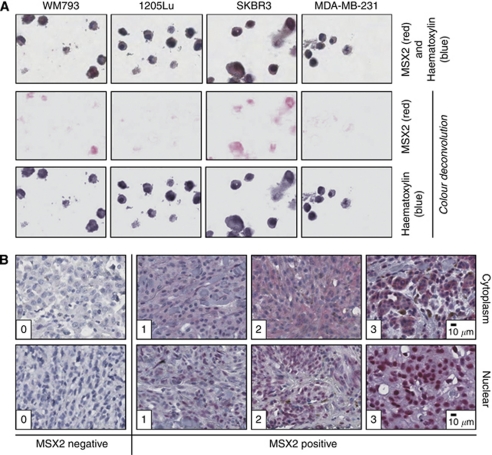
Validation of MSX2 antibody specificity and exemplification of manual scoring system. (**A**) Immunohistochemical staining for MSX2 on FFPE cell lines ( × 20 magnification). For all IHC-based methods, Vector Red Alkaline Phosphatase Substrate Kit I was used for signal detection, producing a red reaction product. The breast cancer cell lines, SKBR3 and MDA-MB-231, were used as positive and negative controls, respectively. A colour deconvolution algorithm and Aperio ImageScope software version 9 were used to separate the red MSX2 signal from the blue haematoxylin counterstain to better visualise the staining results. (**B**) Immunohistochemical staining for MSX2 on melanoma TMA cores, showing sample tumours with manual scores from 0 to 3 for nuclear and cytoplasmic staining ( × 8 magnification). For all further analysis, patients were divided into two groups: MSX2 negative (median score 0) and MSX2 positive (median score 0.5–3), separately for both cytoplasmic and nuclear staining.

To test the applicability of the MSX2 antibody for IHC, a CPA containing FFPE cell lines was stained (Figure 5A). This resulted in a similar staining pattern as observed during western blot analysis (Figure 1A), confirming the specificity of the antibody. Subsequently, the expression of MSX2 protein was assessed in tumour tissues derived from a cohort of melanoma patients (Figure 5B). Staining intensities were graded by two independent pathologists (SH and ML) according to a scale ranging from 0 to 3. Cytoplasmic and nuclear staining were scored separately. For 54 patients (27%), the expression of MSX2 was exclusively cytoplasmic, 76 patients (38%) had both cytoplasmic and nuclear staining, for 27 patients (13.5%) only nuclear MSX2 expression was detectable and 42 tumours (21.5%) did not express MSX2. For all successive analyses, cytoplasmic and nuclear staining were analysed independently. Patients were divided into either an MSX2-negative (median cytoplasmic or nuclear score 0) or an MSX2-positive (median cytoplasmic or nuclear score 0.5–3) subgroup. In total, 70 patients (35%) were classified as MSX2 negative and 130 (65%) as MSX2 positive with regard to cytoplasmic MSX2 expression. According to nuclear staining, 97 tumours (48.5%) were graded as MSX2 negative, while 103 (51.5%) stained positive. Due to core loss or missing histopathological information, 18 tumours were excluded from the analysis.

Next, an association of MSX2 expression with a number of histopathological parameters was investigated (Table 1). Cytoplasmic MSX2 expression was found to correlate significantly with lower T-stages (*P*=0.030). The analysis of nuclear MSX2 expression did not produce any significant correlations.

### Cytoplasmic MSX2 positivity is associated with increased patient survival

**Figure 6:**
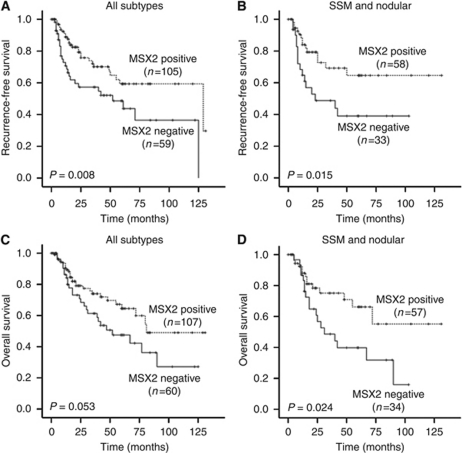
Kaplan–Meier survival analysis of melanoma patients, stratified according to cytoplasmic MSX2 expression. Recurrence-free survival of patients with (**A**) all melanoma subtypes and (**B**) SSM or nodular melanomas only. Overall survival of patients with (**C**) all melanoma subtypes and (**D**) SSM or nodular melanoma only.

In addition to histopathological parameters, patient survival information was available for 167 tumour samples. Kaplan–Meier analysis revealed a significant correlation of cytoplasmic MSX2 expression with increased recurrence-free survival (*P*=0.008; Figure 6A). Subanalyses including superficially spreading and nodular melanomas only showed a significant correlation of cytoplasmic MSX2 positivity with both recurrence-free (*P*=0.015; Figure 6B) and overall survival (*P*=0.024; Figure 6D). No significant correlations were observed for nuclear MSX2 expression and patient outcome (Supplementary Figure 5).

Univariate Cox regression analysis (Table 2) highlighted a significant correlation of positive cytoplasmic MSX2 expression and increased recurrence-free survival (HR 0.489; 95% CI 0.285–0.840; *P*=0.009). To compare the prognostic relevance of MSX2 expression with those of well-established histopathological parameters, such as T-stage and ulceration, multivariate Cox regression analysis was applied. Positive cytoplasmic staining for MSX2 was shown to be an independent prognostic marker for an increase in recurrence-free survival (HR 0.503; 95% CI 0.284–0.893; *P*=0.019). Nuclear MSX2 expression did not produce significant correlations (Supplementary Table 1).

## Discussion

The role of MSX2 in embryonic development has been studied thoroughly throughout the years, while its importance in tumourigenesis is only starting to emerge. Previous studies in pancreatic and breast cancer have painted a diverse picture in respect to this protein. In human pancreatic cancer (Satoh *et al*, 2008), as well as in a mouse mammary epithelial cell line (di Bari *et al*, 2009), ectopic expression of MSX2 was shown to trigger EMT. Histopathological studies on pancreatic cancer samples (Hamada *et al*, 2007; Satoh *et al*, 2008), as well as examination of four infiltrating breast ductal carcinomas (di Bari *et al*, 2009), showed an association between MSX2 and more aggressive disease characteristics. However, in a recent study from our group, MSX2 expression was shown to correlate with good prognosis and over-expression of the protein-induced apoptosis in both a breast cancer and a human mammary epithelial cell line (Lanigan *et al*, 2010).

In an effort to explore the functionality of MSX2 in melanoma, we employed a number of *in vitro* functional assays using the melanoma cell line WM793 and its metastatic derivative 1205Lu (Juhasz *et al*, 1993). Ectopic expression of MSX2 resulted in a loss of cell viability over time and reduced clonogenic activity. An evaluation of Caspase 3/7 activity and cell-cycle distribution suggested a pro-apoptotic role of MSX2 in melanoma cells. No significant impact on cell-cycle progression was detected. In a three-dimensional spheroid model, the pro-apoptotic function of MSX2 appeared to be reduced for WM793 cells and abolished in the case of 1205Lu cells. Instead, a novel aspect of MSX2 protein function, the potent inhibition of cell invasion, became apparent. Similar resistance to apoptosis in three-dimensional cultures has previously been described in the context of drug treatments and can be attributed to enhanced pro-survival signals mediated by the spheroid microenvironment (Smalley *et al*, 2006; Haass *et al*, 2008).

Using western blot analysis, MSX2 could be linked to a number of signalling pathways involved in the regulation of cell survival and invasion. The anti-apoptotic proteins, BCL2 and Survivin, were found to be markedly reduced in response to MSX2 over-expression. BCL2 is expressed in up to 90% of all melanomas (Cerroni *et al*, 1995) and its downregulation using antisense oligonucleotides was shown to be a potent sensitizer for apoptosis in a range of cell lines (Jansen *et al*, 1998; Koty *et al*, 1999). Blocking the activity of the inhibitor of apoptosis protein, Survivin, using a dominant-negative mutant has also been associated with the induction of apoptosis in melanoma cells (Liu *et al*, 2004). Interestingly, Survivin has recently been associated with the regulation of melanoma cell invasion (McKenzie *et al*, 2010) and its downregulation might contribute to the reduction in invasive capacity, observed in the present study.

MSX2 is often mentioned in connection with BMP signalling. Bone morphogenetic proteins are multi-functional growth factors that belong to the transforming growth factor *β* (TGF*β*) superfamily of proteins (Chen *et al*, 2004). Similar to MSX2, they have been ascribed both oncogenic and tumour suppressor activities, depending on their cell type or tissue context (Hsu *et al*, 2005). For instance, BMP4- (Hamada *et al*, 2007) and BMP2- (Ma *et al*, 2005) induced MSX2 expression stimulated EMT in pancreatic cancer and cardiac cushion cells, respectively. On the other hand, BMP4 and MSX2 have been associated with the occurrence of apoptosis in sympathetic neuroblasts (Gomes and Kessler, 2001) and ventricular zone progenitor cells (Israsena and Kessler, 2002). In both cases, the apoptotic effect was not only dependent on the expression of MSX2 but also on p21 (CIP1/WAF1), a well-described downstream target of BMPs. Coincidentally, upregulation of p21 in response to MSX2 over-expression was observed both in breast cancer (Lanigan *et al*, 2010) and in our study on malignant melanoma. This renders a possible role for MSX2 in BMP-mediated apoptosis in these cell types feasible. Interestingly, BMP-induced apoptosis, similar to what has been described earlier for MSX2 over-expression in melanoma, has also been associated with a downregulation of BCL2 and Survivin (Lagna *et al*, 2006). This could be seen as additional, albeit circumstantial, evidence for such an involvement.

Furthermore, BMP4 has been shown to stimulate cleavage of N-Cadherin during neural crest delamination (Shoval *et al*, 2007). In melanoma cells, N-Cadherin is known to promote cell survival and migration (Li *et al*, 2001). Inhibition of N-Cadherin-mediated intercellular interactions using a blocking antibody has been shown previously to increase the level of apoptosis in 1205Lu cells (Li *et al*, 2001). Thus, downregulation of N-Cadherin might be a contributing factor in the induction of apoptosis following MSX2 over-expression. Both N-Cadherin and Survivin have been shown to activate AKT signalling (Li *et al*, 2001; McKenzie *et al*, 2010). However, MSX2-induced downregulation of these proteins did not result in decreased levels of p-AKT, indicating the activation of an MSX2-specific signalling pathway or compensation through other proteins. Similarly, ectopic expression of MSX2 in breast cancer cells was shown to elevate p-ERK levels (Lanigan *et al*, 2010), yet no such correlation could be observed in melanoma. Interestingly, in breast cancer, ERK activation was less pronounced in MDA-MB-231, a cell line harbouring KRAS G38A and BRAF G464V mutations, compared with the non-transformed epithelial cell line MCF10a. Both WM793 and 1205Lu cells express mutant BRAF V600E that might mask additional, MSX2-mediated activation of ERK. Moreover, future studies will clarify a direct or indirect role of the above-mentioned proteins in MSX2-induced apoptosis and the regulation of cell invasion.

In addition, a comprehensive cohort of 218 primary melanoma samples was screened for the clinical relevance of MSX2 protein expression. Cytoplasmic MSX2 expression was significantly associated with lower T-stages and prolonged recurrence-free survival. Nuclear MSX2 expression showed no significant correlations. This suggests that the primary, clinically beneficial function of MSX2 is not coupled with DNA binding and direct transcriptional regulation in the nucleus. Instead, a mechanism based on protein–protein interactions is proposed. This is supported by previous findings that demonstrate that the transcriptional activity of MSX2 does not depend on its homeodomain DNA binding sites but can be directed to heterologous promoters, most likely through interactions with other proteins (Catron *et al*, 1996). In similar regard, the formation of nuclear heterodimers with the homeobox proteins MIZ1 and DLX5 and their impact on DNA binding are well described (Satoh *et al*, 2004). Here, we propose a slightly different mechanism where MSX2 forms heterodimers in the cytoplasm to sequester its binding partner from another location or to form an active signalling complex. However, future studies are needed to shed light onto this concept and the spatial relevance of MSX2 expression. During *in vitro* characterisation studies, MSX2 was primarily located to the nucleus of melanoma cells with some cytoplasmic staining being detectable. Further *in vitro* models with defined local MSX2 expression will help to clarify the relevance of *in vitro* results for clinical outcome.

Overall, *in vitro* over-expression of MSX2 led to the induction of apoptosis and a clear reduction in melanoma cell invasiveness. A number of signalling proteins were shown to be altered by MSX2 over-expression, including BCL2, Survivin and N-Cadherin. In addition, MSX2 was shown to be associated with good prognosis in melanoma. Cytoplasmic expression of the protein correlated significantly with longer recurrence-free and overall survival. In addition, multivariate Cox regression analysis could establish cytoplasmic MSX2 expression as an independent prognostic factor for increased recurrence-free survival.

## Figures and Tables

**Table 1:** Association of cytoplasmic and nuclear MSX2 with histopathological parameters

		**Cytoplasmic MSX2**			**Nuclear MSX2**		
**Variable**	***N***	**MSX2 negative, *N* (%)**	**MSX2 positive, *N* (%)**	***P*-value^a^**	**MSX2 negative, *N* (%)**	**MSX2 positive, *N* (%)**	***P*-value** a
*T-stage*	189			**0.030**			0.655
T1		12 (18)	39 (32)		24 (26)	27 (28)	
T2–T4		56 (82)	82 (68)		70 (74)	68 (72)	
							
*Clark level*	182			0.088			0.903
1–3		28 (43)	67 (58)		45 (52)	50 (53)	
4–5		37 (57)	50 (42)		42 (48)	45 (47)	
							
*Subtype*	190			0.658			0.126
SSM		19 (28)	39 (32)		35 (37)	23 (24)	
Nodular		21 (31)	31 (25)		22 (23)	30 (31)	
Other		27 (40)	53 (43)		37 (39)	43 (45)	
							
*Ulceration*	177			0.115			0.492
Absent		41 (64)	85 (75)		62 (69)	64 (74)	
Present		23 (36)	28 (25)		28 (31)	23 (26)	
							
*Site*	188			0.576			0.532
Head and neck		15 (22)	38 (31)		22 (23)	31 (33)	
Trunk		13 (19)	20 (17)		17 (18)	16 (17)	
Upper limb		12 (18)	19 (16)		17 (18)	14 (15)	
Lower limb		28 (41)	43 (36)		38 (40)	33 (35)	

Abbreviations: *N*=number of patients; SSM=superficial spreading melanoma.

a *χ*^2^-test. *P*-values <0.05 is shown in bold.

**Table 2:** Cox regression analysis of recurrence-free survival and overall survival (entire cohort)

	**Univariate**			**Multivariate^a^**		
**Prognostic factor**	**HR**	**(95% CI)**	***P*-value**	**HR**	**(95% CI)**	***P*-value**
*Recurrence-free survival*						
Cytoplasmic MSX2 (positive *vs* negative, ref)	0.489	0.285–0.840	**0.009**	0.503	0.284–0.893	**0.019**
T-stage (T2–T4 *vs* T1, ref)	7.466	2.689–20.739	**<0.001**	6.372	1.955–20.767	**0.002**
Ulceration (positive *vs* negative, ref)	3.267	1.846–5.781	**<0.001**	2.653	1.464–4.809	**0.001**
						
*Overall survival*						
Cytoplasmic MSX2 (positive *vs* negative, ref)	0.594	0.348–1.014	0.059	0.653	0.376–1.136	0.132
T-stage (T2–T4 *vs* T1, ref)	9.696	3.021–31.117	**<0.001**	10.060	2.419–41.841	**0.002**
Ulceration (positive *vs* negative, ref)	3.528	2.021–6.160	**<0.001**	2.900	1.610–5.223	**<0.001**

Abbreviations: CI=confidence interval; HR=hazard ratio; ref=referent group.

a Adjusted for all other variables in the subsection. *P*-values <0.05 are shown in bold.
