# Supplementary material for: Functional and prognostic relevance of the homeobox protein MSX2 in malignant melanoma
Source: Br J Cancer. 2011 Jul 5;105(4):565–74. doi: 10.1038/bjc.2011.249 (PMC3170959; doi:10.1038/bjc.2011.249)
Supplement: Supplementary Information [file bjc2011249x1.doc]

**Supplementary Information**

**Supplementary Materials and Methods**

**Soft agar colony formation**

Six-well plates were coated with 0.5% (w/v) agarose in growth media and 0.35% (w/v) agarose in media containing 50 000 cells/well layered on top. Two ml of normal growth media was added per well and replaced weekly. After three weeks, colonies were counted using a standard bright field microscope. The number of colonies in four randomised microscopic fields (10x magnification) per assay condition was determined.

**Supplementary Figures**

Supplementary Figure 1: Determination of spheroid invasiveness. Images were analysed using Aperio ImageScope software version 10 (Aperio Technologies, Vista, CA). An area of interest was drawn around the core spheroid structure and the number of pixels within this area determined. The invaded area was measured using a positive pixel count algorithm. Next, the core spheroid structure was idealised as a circle with radius r (calculated from the core spheroid area). A band with radius r was drawn around the idealised core spheroid structure and the total number of pixels in this band calculated (100% reference area). Finally, the invaded area pixel count was normalised to the 100% reference area.

**Supplementary Figure 2:** Functional characterisation of DOX-induced (+MSX2) vs. non-induced control cells (-MSX2) in two-dimensional cultures of WM793 and 1205Lu cells (sample results including all control cell lines). **(a)** Evaluation of cell viability over time using MTT assays. Significance was generated from the average signal change between consecutive days in three independent experiments. **(b)** Determination of clonogenic cell survival. **(c)** Assessment of caspase 3/7 activation using a bioluminescence-based Caspase-Glo 3/7 assay system. Luminescence readings were normalised to the total cell number and displayed relative to non-induced control cells. **(d)** Sub-G1 cell cycle population as detected via PI staining followed by flow cytometric analysis. Error bars represent the standard error of the mean within internal replicates. Significance levels were determined using an unpaired, two-tailed Student’s t-test. KL, cells infected with KRAB and LLCIEP (empty vector) encoding virus; KM, cells infected with KRAB and MSX2 encoding virus. p-Values refer to KM -/+ DOX results. Significance levels: * p<0.05, ** p<0.01, *** p<0.001.

**Supplementary Figure 3:** Cell cycle analysis in WM793 and 1205Lu cells in response to MSX2-over-expression. Representative histograms after PI staining and flow analysis of DOX-induced (+ MSX2) vs. non-induced control cells (- MSX2) including the Sub-G1 population of the cell cycle in **(a)** WM793 cells and **(c)** 1205Lu cells. Representative histograms after PI staining and flow analysis including viable cells only in **(b)** WM793 cells and **(d)** 1205Lu cells.

**Supplementary Figure 4:** Dose-dependent effect of MSX2 expression in WM793 cells. **(a)** Western blot analysis of MSX2 protein levels in response to a gradient of DOX (as specified in panel **(c)**). β-Actin levels were used to assess total protein loading. Exposure time was adjusted to strongest signal to avoid over-exposure. **(b)** Cell cycle analysis by propidium iodide staining and flow cytometry-based evaluation. Values represent the percentage of cells in Sub-G1, an indicator for cellular apoptosis. **(c)** Soft agar colony formation assay to test for clonogenic survival/proliferation of cells in the absence of anchorage. Significance levels: * p<0.05, ** p<0.01, *** p<0.001.

**Supplementary Figure 5:** Kaplan-Meier survival analysis of melanoma patients, stratified according to nuclear MSX2 expression. Recurrence-free survival of patients with (a) all melanoma subtypes and (b) SSM or nodular melanomas only. Overall survival of patients with (c) all melanoma subtypes and (d) SSM or nodular melanoma only.

**Supplementary Table**

Supplementary Table 1: Cox regression analysis of nuclear MSX2 expression and recurrence-free survival or overall survival (entire cohort).

| **Prognostic Factor** | **Univariate** | | |  | **Multivariate^1^** | | |
| --- | --- | --- | --- | --- | --- | --- | --- |
|  | HR | (95% CI) | p-value |  | HR | (95% CI) | p-value |
| **Recurrence Free Survival** |  |  |  |  |  |  |  |
| Nuclear MSX2 (pos. vs. neg., ref) | 0.973 | 0.570-1.660 | 0.919 |  | 0.807 | 0.453-1.425 | 0.460 |
| T-Stage (T2-T4 vs. T1, ref) | 7.466 | 2.689-20.739 | **<0.001** |  | 6.868 | 2.110-22.362 | **0.001** |
| Ulceration (pos. vs. neg., ref) | 3.267 | 1.846-5.781 | **<0.001** |  | 2.767 | 1.532-4.998 | **0.001** |
| **Overall Survival** |  |  |  |  |  |  |  |
| Nuclear MSX2 (pos. vs neg., ref) | 1.003 | 0.587-1.714 | 0.990 |  | 0.912 | 0.525-1.587 | 0.746 |
| T-Stage (T2-T4 vs. T1, ref) | 9.696 | 3.021-31.117 | **<0.001** |  | 10.350 | 2.486-43.027 | **0.001** |
| Ulceration (pos. vs. neg., ref) | 3.528 | 2.021-6.160 | **<0.001** |  | 2.920 | 1.627-5.242 | **<0.001** |

^1^Adjusted for all other variables in the subsection; ref, referent group; HR, hazard ratio; CI, confidence interval
